# Validation of Ecological Sustained Attention Test for Schizophrenia

**DOI:** 10.1192/j.eurpsy.2025.423

**Published:** 2025-08-26

**Authors:** R. Komemi, T. Goldberger, A. Ariel, A. Zagory, M. Nahum, L. Lipskaya-Velikovsky

**Affiliations:** 1 Hebrew University, Jerusalem, Israel

## Abstract

**Introduction:**

Sustained attention deficit is a core feature of schizophrenia, however, its between-subjects and within-subject fluctuations are little understood. Moreover, although the pertinence of sustained attention for daily functioning has been widely discussed in the literature, research has not consistently demonstrated this association in schizophrenia. One possible reason is a use with non-ecological tasks for evaluation of SA, which may not fully capture real-world attentional demands. Indeed, existing tools demonstrate low ecological validity.

**Objectives:**

This study aimed to develop daily-life task-based test of SA - Ecological Sustained Attention Task (Eco-SAT), and investigate its reliability and criterion, construct, and ecological validity in schizophrenia.

**Methods:**

Eco-SAT was developed based on well-established CPT paradigm (320 trials presented for 500-3000ms, 12 min) simulating vacuum cleaning task. Twenty-one individuals with schizophrenia (age: M=42, SD=12.5; female: N=12, 57%) and 34 matched by age and gender healthy controls completed the Eco-SAT, non-ecological CPT, measures of cognition (MATRICS consensus cognitive battery: BACS, TMT, RBVMT & CFT), schizophrenia symptoms, and daily functioning using Observed Tasks of Daily Living test (OTDL, functional capacity) and Adults Subjective Assessment of Participation in daily life in an interchangeable order during one session.

**Results:**

Eco-SAT demonstrated excellent test-retest reliability for Average RT (ICC = 0.84). Schizophrenia patients performed Eco-SAT significantly worse than controls on Average RT (U=545, p < 0.01), and variance RT (U=518, p < 0.01). Eco-SAT showed correlations with non-ecological SA task (all parameters; 0.45<r<0.75, p<.001), MATRICS sub-tests of BACS (d prime, average RT, RT variance: -0.54<r<0.3, p<.5), TMT-B (average RT: r=0.3, p<.05), RBVMT (average RT, RT variance: -0.5<r<-0.4, p<.01), and CFT (average RT, RT variance:-0.35<r<-0.34, p<.001), positive schizophrenia symptoms (hit rate: r=0.3, p<.05) and the OTDL (RT variance, d prime, hit rate: -0.35<r<0.3,p < .05).

**Conclusions:**

The study provides initial evidence of the psychometric properties of the Eco-SAT, an ecological measure of sustained attention in schizophrenia. Criterion validity was established for all Eco-SAT indices, while reliability, construct validity, and ecological validity were demonstrated for specific indices. The results suggest that average RT and RT variance are the most trustworthy indices. Although further research is required, these findings indicate that this ecological measure may offer a more accurate way to assess attentional deficits in real-world activities, enhancing our understanding of attentional mechanisms in schizophrenia for both research and clinical applications.

**Disclosure of Interest:**

None Declared

